# Supplementary material for: The RNA secondary structure of androgen receptor-FL and V7 transcripts reveals novel regulatory regions
Source: Nucleic Acids Res. 2024 Mar 30;52(11):6596–613. doi: 10.1093/nar/gkae220 (PMC11194067; doi:10.1093/nar/gkae220)
Supplement: gkae220_Supplemental_Files [file gkae220_supplemental_files.zip › Supplementary File S5.zip!/Supplementary File S5/FL_CaCo.zip!/FL_CaCo/AR_M23_CDS_1.fold.R2R.sto.pdf]

AR_M23_CDS_1

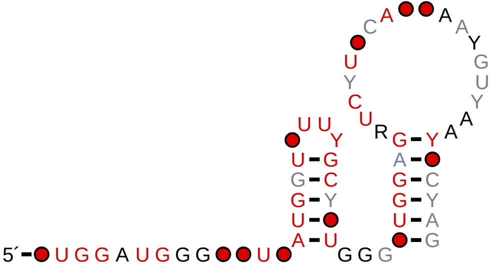
5´
UGGAUGGG
U
A
U
G
G
U
UUY
G
C
Y
U
GGG
G
A
G
G
U
G
Y
C
Y
A
G
R
UCUYUCA
AAYGUYAA
